# Comparing formative and summative simulation-based assessment in undergraduate nursing students: nursing competency acquisition and clinical simulation satisfaction

**DOI:** 10.1186/s12912-021-00614-2

**Published:** 2021-06-08

**Authors:** Oscar Arrogante, Gracia María González-Romero, Eva María López-Torre, Laura Carrión-García, Alberto Polo

**Affiliations:** Fundación San Juan de Dios, Centro de Ciencias de la Salud San Rafael, Universidad de Nebrija, Paseo de La Habana, 70, 28036 Madrid, Spain

**Keywords:** Clinical competence, High Fidelity simulation training, Learning, Nursing students

## Abstract

**Background:**

Formative and summative evaluation are widely employed in simulated-based assessment. The aims of our study were to evaluate the acquisition of nursing competencies through clinical simulation in undergraduate nursing students and to compare their satisfaction with this methodology using these two evaluation strategies.

**Methods:**

Two hundred eighteen undergraduate nursing students participated in a cross-sectional study, using a mixed-method. MAES© (self-learning methodology in simulated environments) sessions were developed to assess students by formative evaluation. Objective Structured Clinical Examination sessions were conducted to assess students by summative evaluation. Simulated scenarios recreated clinical cases of critical patients. Students´ performance in all simulated scenarios were assessed using checklists. A validated questionnaire was used to evaluate satisfaction with clinical simulation. Quantitative data were analysed using the IBM SPSS Statistics version 24.0 software, whereas qualitative data were analysed using the ATLAS-ti version 8.0 software.

**Results:**

Most nursing students showed adequate clinical competence. Satisfaction with clinical simulation was higher when students were assessed using formative evaluation. The main students’ complaints with summative evaluation were related to reduced time for performing simulated scenarios and increased anxiety during their clinical performance.

**Conclusion:**

The best solution to reduce students’ complaints with summative evaluation is to orient them to the simulated environment. It should be recommended to combine both evaluation strategies in simulated-based assessment, providing students feedback in summative evaluation, as well as evaluating their achievement of learning outcomes in formative evaluation.

## Background

Clinical simulation methodology has increased exponentially over the last few years and has gained acceptance in nursing education. Simulation-based education (SBE) is considered an effective educational methodology for nursing students to achieve the competencies needed for their professional future [1–5]. In addition, simulation-based educational programs have demonstrated to be more useful than traditional teaching methodologies [4, 6]. As a result, most nursing faculties are integrating this methodology into their study plans [7]. SBE has the potential to shorten the learning curve for students, increase the fusion between theoretical knowledge and clinical practice, establish deficient areas in students, develop communication and technical skills acquisition, improve patient safety, standardise the curriculum and teaching contents, and offer observations of real-time clinical decision making [5, 6, 8, 9].

SBE offers an excellent opportunity to perform not only observed competency-based teaching, but also the assessment of these competencies. Simulated-based assessment (SBA) is aimed at evaluating various professional skills, including knowledge, technical and clinical skills, communication, and decision-making; as well as higher-order competencies such as patient safety and teamwork skills [1–4, 10]. Compared with other traditional assessment methods (i.e. written or oral test), SBA offers the opportunity to evaluate the actual performance in an environment similar to the ‘real’ clinical practice, assess multidimensional professional competencies, and present standard clinical scenarios to all students [1–4, 10].

The main SBA strategies are formative and summative evaluation. Formative evaluation is conducted to establish students’ progression during the course [11]. This evaluation strategy is helpful to educators in improving students’ deficient areas and testing their knowledge [12]. Employing this evaluation strategy, educators give students feedback about their performance. Subsequently, students self-reflect to evaluate their learning and determine their deficient areas. In this sense, formative evaluation includes an ideal phase to achieve the purposes of strategy: the debriefing [13]. International Nursing Association for Clinical Simulation and Learning (INACSL) defines debriefing as a reflective process immediately following the simulation-based experience where ‘participants explore their emotions and question, reflect, and provide feedback to one another’. Its aim is ‘to move toward assimilation and accommodation to transfer learning to future situations’ [14]. Therefore, debriefing is a basic component for learning to be effective after the simulation [15, 16]. Furthermore, MAES© (according to its Spanish initials of self-learning methodology in simulated environments) is a clinical simulation methodology created to perform formative evaluations [17]. MAES© allows evaluating specifically nursing competencies acquired by several nursing students at the same time. MAES© is structured through the union of other active learning methodologies such as self-directed learning, problem-based learning, peer education and simulation-based learning. Specifically, students acquire and develop competencies through self-directed learning, as they voluntarily choose competencies to learn. Furthermore, this methodology encourages students to be the protagonists of their learning process, since they can choose the case they want to study, design the clinical simulation scenario and, finally, actively participate during the debriefing phase [17]. This methodology meets all the requirements defined by the INACSL Standards of Best Practice [18]. Compared to traditional simulation-based learning (where simulated clinical scenarios are designed by the teaching team and led by facilitators), the MAES© methodology (where simulated clinical scenarios are designed and led by students) provides students nursing a better learning process and clinical performance [19]. Currently, the MAES© methodology is used in clinical simulation sessions with nursing students in some universities, not only in Spain but also in Norway, Portugal and Brazil [20].

In contrast, summative evaluation is used to establish the learning outcomes achieved by students at the end of the course [11]. This evaluation strategy is helpful to educators in evaluating students’ learning, the competencies acquired by them and their academic achievement [12]. This assessment is essential in the education process to determine readiness and competence for certification and accreditation [10, 21]. Accordingly, Objective Structured Clinical Examination (OSCE) is commonly conducted in SBA as a summative evaluation to evaluate students’ clinical competence [22]. Consequently, OSCE has been used by educational institutions as a valid and reliable method of assessment. OSCE most commonly consists of a ‘round-robin’ of multiple short testing stations, in each of which students must demonstrate defined clinical competencies, while educators evaluate their performance according to predetermined criteria using a standardized marking scheme, such as checklists. Students must rotate through these stations where educators assess students’ performance in clinical examination, technical skills, clinical judgment and decision-making skill during the nursing process [22, 23]. This strategy of summative evaluation incorporates actors performing as simulated patients. Therefore, OSCE allows assessing students’ clinical competence in a real-life simulated clinical environment. After simulated scenarios, this evaluation strategy provides educators with an opportunity to give students constructive feedback according to their achieved results in the checklist [10, 21–23].

Despite both evaluation strategies are widely employed in SBA, there is scarce evidence about the possible differences in satisfaction with clinical simulation when nursing students are assessed using formative and summative evaluation. Considering the high satisfaction with the formative evaluation perceived by our students during the implementation of the MAES© methodology, we were concerned if this satisfaction would be similar using the same simulated clinical scenarios through a summative evaluation. Additionally, we were concerned about the reasons why this satisfaction would be different using both strategies of SBA. Therefore, the aims of our study were to evaluate the acquisition of nursing competencies through clinical simulation methodology in undergraduate nursing students, as well as to compare their satisfaction with this methodology using two strategies of SBA, such as formative and summative evaluation. In this sense, our research hypothesis is that both strategies of SBA are effective in acquiring nursing competencies, but student satisfaction with the formative evaluation is higher than with the summative evaluation.

## Methods

### Study design and setting

A descriptive cross-sectional study using a mixed-method and analysing both quantitative and qualitative data. The study was conducted from September 2018 to May 2019 in a University Centre of Health Sciences in Madrid (Spain). This centre offers Physiotherapy and Nursing Degrees.

### Participants

The study included 3rd-year undergraduate students (106 students participated in MAES© sessions within the subject ‘Nursing care for critical patients’) and 4th-year undergraduate students (112 students participated in OSCE sessions within the subject ‘Supervised clinical placements – Advanced level’) in Nursing Degree. It should be noted, 4th-year undergraduate students had completed all their clinical placements and they had to approve OSCE sessions to achieve their certification.

### Clinical simulation sessions

To assess the clinical performance of 3rd-year undergraduate students using formative evaluation, MAES© sessions were conducted. This methodology consists of 6 elements in a minimum of two sessions [17]: Team selection and creation of group identity (students are grouped into teams and they create their own identity), voluntary choice of subject of study (each team will freely choose a topic that will serve as inspiration for the design of a simulation scenario), establishment of baseline and programming skills to be acquired through brainstorming (the students, by teams, decide what they know about the subject and then what they want to learn from it, as well as the clinical and non- technical skills they would like to acquire with the case they have chosen), design of a clinical simulation scenario in which the students practice the skills to be acquired (each team commits to designing a scenario in the simulation room), execution of the simulated clinical experience (another team, different from the one that has designed the case, will enter the high-fidelity simulation room and will have a simulation experience), and finally debriefing and presentation of the acquired skills (in addition to analysing the performance of the participants in the scenario, the students explain what they learned during the design of the case and look for evidence of the learning objectives).

Alternatively, OSCE sessions were developed to assess the clinical performance of 4th-year undergraduate students using summative evaluation. Both MAES© and OSCE sessions recreated critically ill patients with diagnoses of Exacerbation of Chronic Obstructive Pulmonary Disease (COPD), acute coronary syndrome haemorrhage in a postsurgical, and severe traumatic brain injury.

It should be noted that the implementation of all MAES© and OSCEs sessions followed the Standards of Best Practice recommended by the INACSL [14, 24–26]. In this way, all the stages included in a high-fidelity session were accomplished: pre-briefing, briefing, simulated scenario, and debriefing. Specifically, a session with all nursing students was carried out 1 week before the performance of OSCE stations to establish a safe psychological learning environment and familiarize students with this summative evaluation. In this pre-briefing phase, we implemented several activities based on practices recommended by the INACSL Standards Committee [24, 25] and Rudolph, Raemer, and Simon [27] for establishing a psychologically safe context. Although traditional OSCEs do not usually include the debriefing phase, we decided to include this phase in all OSCEs carried out in our university centre, since we consider this phase is quite relevant to nursing students’ learning process and their imminent professional career.

Critically ill patient’s role was performed by an advanced simulator mannequin (NursingAnne® by Laerdal Medical AS) in all simulated scenarios. A confederate (a health professional who acts in a simulated scenario) performed the role of a registered nurse or a physician who could help students as required. Occasionally, this confederate could perform the role of a relative of a critically ill patient. Nursing students formed work teams of 2–3 students in all MAES© and OSCE sessions. Specifically, each work team formed in MAES© sessions received a brief description of simulated scenario 2 months before and students had to propose 3 NIC (Nursing Interventions Classification) interventions [28], and 5 related nursing activities with each of them, to resolve the critical situation. In contrast, the critical situation was presented to each work team formed in OSCE sessions for 2 min before entering the simulated scenario. During all simulated experiences, professors were monitoring and controlling the simulation with a sophisticated computer program in a dedicated control room. All simulated scenarios lasted 10 min.

After each clinical simulated scenario was concluded, a debriefing was carried out to give students feedback about their performance. Debriefings in MAES© sessions were conducted according to the Gather, Analyse, and Summarise (GAS) method, a structured debriefing model developed by Phrampus and O’Donnell [29]. According to this method, the debriefing questions used were: *What went well during your performance?; What did not go so well during your performance?; How can you do better next time?*. Additionally, MAES© includes an expository phase in debriefings, where the students who performed the simulated scenario establish the contributions of scientific evidence about its resolution [17]. Each debriefing lasted 20 min in MAES© sessions. In contrast, debriefings in OSCE sessions lasted 10 min and they were carried out according to the Plus-Delta debriefing tool [30], a technique recommended when time is limited. Consequently, the debriefing questions were reduced to two questions: *What went well during your performance?; What did not go so well during your performance?*. Within these debriefings, professors communicated to students the total score obtained in the appropriate checklist. Each debriefing lasted 10 min in OSCE sessions. After all debriefings, students completed the questionnaires to evaluate their satisfaction with clinical simulation. In OSCE sessions, students had to report their satisfaction only with the scenario performed, which took part in a series of clinical stations.

In summary, Table 1 shows the required elements for formative and summative evaluation according to the Standards of Best Practice for participant evaluation recommended by the INACSL [18]. It should be noted that our MAES© and OSCE sessions accomplished these required elements.

**Table 1:** Required elements for formative and summative evaluation according to the Standards of Best Practice for participant evaluation recommended by the International Nursing Association for Clinical Simulation and Learning (INACSL, 2016)

Formative evaluation	Summative evaluation
Formative evaluation is conducted to: • Monitor progress toward achieving outcomes. • Provide ongoing formative feedback. • Support participant’s clinical competencies. • Identify and close gaps in knowledge and skills. • Assess readiness for real-world experiences. • Facilitate teaching and learning.	Summative evaluation is conducted: • At a discrete point in time (i.e., at the end of a course or certain time period). • In a safe learning environment. • After orientation to the environment and equipment. • Appropriate level of fidelity necessary to achieve the participant outcomes. • Utilizing a standardized format and scoring methods (i.e., utilizing a standardized scenario that includes information on when to cue, scenario length of time, and other scenario details). • With a video recording of the evaluation to allow review by multiple trained evaluators
Requires formally trained facilitators (see INACSL Standard: Facilitation).	Use a theoretically based method to determine passing or cut scores where appropriate.
Use small group ratio, ideally a minimum ratio of one facilitator per three to five students.	Select a valid and reliable instrument.
	Provide rater training for observation-based evaluation.
	Establish interrater reliability when more than one rater required.
	Inform participants in advance of the evaluation.
	Provide summative feedback to participant about achievement of outcomes.

### Instruments

#### Clinical performance

Professors assessed students’ clinical performance using checklists (‘Yes’/‘No’). In MAES© sessions, checklists were based on the 5 most important nursing activities included in the NIC [28] selected by nursing students. Table 2 shows the checklist of the most important NIC interventions and its related nursing activities selected by nursing students in the Exacerbation of Chronic Obstructive Pulmonary Disease (COPD) simulated scenario. In contrast, checklists for evaluating OSCE sessions were based on nursing activities selected by consensus among professors, registered nurses, and clinical placement mentors. Nursing activities were divided into 5 categories: nursing assessment, clinical judgment/decision-making, clinical management/nursing care, communication/interpersonal relationships, and teamwork. Table 3 shows the checklist of nursing activities that nursing students had to perform in COPD simulated scenario. During the execution of all simulated scenarios, professors checked if the participants perform or not the nursing activities selected.

**Table 2:** Formative evaluation: Checklist of the most important NIC interventions and its related nursing activities [28] selected by nursing students in Exacerbation of Chronic Obstructive Pulmonary Disease (COPD) simulated scenario

**[3350] Respiratory Monitoring**	YES	NO
Monitor rate, rhythm, depth, and effort of respirations		
Auscultate lung sounds after treatments to note results		
Note changes in SaO_2_, SvO_2_, end tidal CO_2_, and ABG values, as appropriate		
Monitor for increased restlessness, anxiety, and air hunger		
Institute respiratory therapy treatments (e.g., nebuliser), as needed		
**[3302] Mechanical Ventilation Management: Noninvasive**	YES	NO
Monitor for conditions indicating the appropriateness of noninvasive ventilation support (e.g., acute exacerbations of COPD)		
Consult with other health care personnel in selection of a noninvasive ventilator type (e.g., pressure limited [bilevel positive airway pressure], volume-cycled flow-limited, or CPAP)		
Instruct the patient and family about the rationale and expected sensations associated with the use of noninvasive mechanical ventilators and devices		
Place the patient in semi-Fowler position		
Apply noninvasive device, assuring adequate fit and avoidance of large air leaks (take particular care with edentulous or bearded patients)		
**[5820] Anxiety Reduction**	YES	NO
Use a calm, reassuring approach		
Clearly state expectations for patient’s behaviour		
Explain all procedures, including sensations likely to be experienced during the procedure		
Provide factual information concerning diagnosis, treatment, and prognosis		
Stay with the patient to promote safety and reduce fear

**Table 3:** Summative evaluation: Checklist of nursing activities performed by nursing students in Exacerbation of Chronic Obstructive Pulmonary Disease (COPD) simulated scenario

NURSING ASSESSMENT (20 points)	YES	NO	Points
They perform a focused respiratory exploration through appropriate pulmonary auscultation (5 points)			
They recognise correctly signs and symptoms of respiratory distress, including SaO_2_ (5 points)			
They assess correctly haemodynamic signs and symptoms (5 points)			
They interpret correctly the complementary tests ordered by the physician (5 points)			
CLINICAL JUDGEMENT AND DECISION-MAKING (20 points)	YES	NO	Points
They diagnose correctly the patient’s clinical situation (5 points)			
They prioritise adequately nursing interventions (5 points)			
They re-evaluate the patient according to nursing assessment (5 points)			
They apply the appropriate treatment for respiratory distress at the right time (5 points)			
CLINICAL MANAGEMENT AND NURSING CARE (30 points)	YES	NO	Points
Handwashing (2.5 points)			
Use of gloves (2.5 points)			
They place the patient in semi-Fowler position (2.5 points)			
Proper pulse oximeter placement (2.5 points)			
Proper EEG electrodes placement (2.5 points)			
Proper blood pressure cuff placement (2.5 points)			
They apply correctly the adequate oxygen therapy according to nursing assessment (2.5 points)			
They call a physician (2.5 points)			
They follow properly physician instructions (2.5 points)			
They administer correctly the prescribed medication (2.5 points)			
They evaluate the patient’s response to the medical treatment administered (2.5 points)			
They perform correctly the complementary test ordered by the physician (2.5 points)			
COMMUNICATION AND INTERPERSONAL RELATIONSHIPS (15 points)	YES	NO	Points
They introduce themselves to the patient (3 points)			
They reduce the patient’s anxiety (3 points)			
They show empathy, active listening and respect when they communicate with the patient and/or family (3 points)			
Appropriate communication with the physician (3 points)			
Appropriate communication among team members (3 points)			
TEAMWORK (15 points)	YES	NO	Points
Appropriate coordination among team members and they demonstrate an effective teamwork (15 points)			
	TOTAL		

#### Clinical simulation satisfaction

To determine satisfaction with clinical simulation perceived by nursing students, the Satisfaction Scale Questionnaire with High-Fidelity Clinical Simulation [31] was used after each clinical simulation session. This questionnaire consists of 33 items with a 5-point Likert scale ranging from ‘strongly disagree’ to ‘totally agree’. These items are divided into 8 scales: simulation utility, characteristics of cases and applications, communication, self-reflection on performance, increased self-confidence, relation between theory and practice, facilities and equipment and negative aspects of simulation. Cronbach’s α values for each scale ranged from .914 to .918 and total scale presents satisfactory internal consistency (Cronbach’s α value = .920). This questionnaire includes a final question about any opinion or suggestion that participating students wish to reflect after the simulation experience.

### Data analysis

Quantitative data were analysed using IBM SPSS Statistics version 24.0 software for Windows (IBM Corp., Armonk, NY, USA). Descriptive statistics were calculated to interpret the results obtained in demographic data, clinical performance, and satisfaction with clinical simulation. The dependent variables after the program in the two groups were analyzed using independent t-tests. The differences in the mean changes between the two groups were analyzed using an independent t-test. Cohen’s d was calculated to analyse the effect size for t-tests. Statistical tests were two-sided (α = 0.05), so the statistical significance was set at 0.05. Subsequently, all students’ opinions and comments were analysed using the ATLAS-ti version 8.0 software (Scientific Software Development GmbH, Berlin, Germany). All the information contained in these qualitative data were stored, managed, classified and organized through this software. All the reiterated words, sentences or ideas were grouped into themes using a thematic analysis [32]. It should be noted that the students’ opinions and comments were preceded by the letter ‘S’ (student) and numerically labelled.

## Results

A total of 218 nursing students participated in the study (106 students were trained through MAES© sessions, whereas 112 students were assessed through OSCE sessions). The age of students ranged from 20 to 43 years (mean = 23.28; SD = 4.376). Most students were women (*n* = 184; 84.4%).

In formative evaluation, professors checked 93.2% of students selected adequately both NIC interventions and its related nursing activities for the resolution of the clinical simulated scenario. Subsequently, these professors checked 85.6% of students, who participated in each simulated scenario, performed the nursing activities previously selected by them. In summative evaluation, students obtained total scores ranged from 65 to 95 points (mean = 7.43; SD = .408).

Descriptive data for each scale of satisfaction with clinical simulation questionnaire, t-test, and effect sizes (d) of differences between two evaluation strategies are shown in Table 4. Statistically significant differences were found between two evaluation strategies for all scales of the satisfaction with clinical simulation questionnaire. Students´ satisfaction with clinical simulation was higher for all scales of the questionnaire when they were assessed using formative evaluation, including the ‘negative aspects of simulation’ scale, where the students perceived fewer negative aspects. The effect size of these differences was large (including the total score of the questionnaire) (Cohen’s d values > .8), except for the ‘facilities and equipment’ scale, which effect size was medium (Cohen’s d value > .5) [33].

**Table 4:** Descriptive data, t-test and effect sizes (d) of differences between two evaluation strategies for scales of clinical simulation satisfaction (*n* = 218)

Scale	Formative evaluation (***n*** = 106)	Summative evaluation (***n*** = 112)	t_**(1,217)**_	Sig.	Effect size (d)
	Mean (SD)	Mean (SD)			
Simulation utility	56.59 (5.584)	52.67 (10.109)	21.71	.001	3.925
Characteristics of cases and application	18.57 (1.487)	16.74 (2.690)	27.84	<.001	1.825
Communication	14.36 (1.244)	12.98 (2.379)	42.13	<.001	1.376
Self-reflection on performance	14.28 (1.119)	12.73 (2.438)	35.84	<.001	1.551
Increased self-confidence	13.72 (1.378)	11.71 (3.071)	42.87	<.001	2.003
Relation between theory and practice	13.78 (1.345)	11.71 (2.447)	41.43	<.001	2.069
Facilities and equipment	12.20 (1.775)	11.58 (2.225)	4.29	.024	.618
Negative aspects of simulation	3.73 (1.231)	4.77 (.849)	12.09	<.001	-.947
Total score	147.23 (9.977)	134.61 (21.955)	35.10	<.001	12.619

Table 5 shows specifically descriptive data, t-test, and effect sizes (d) of differences between both evaluation strategies for each item of the clinical simulation satisfaction questionnaire. Statistically significant differences were found between two evaluation strategies for all items of the questionnaire, except for items ‘I have improved communication with the family’, ‘I have improved communication with the patient’, and ‘I lost calm during any of the cases’. Students´ satisfaction with clinical simulation was higher in formative evaluation sessions for most items, except for item ‘simulation has made me more aware/worried about clinical practice’, where students informed being more aware and worried in summative evaluation sessions. Most effect sizes of these differences were small or medium (Cohen’s d values ranged from .238 to .709) [33]. The largest effect sizes of these differences were obtained for items ‘timing for each simulation case has been adequate’ (d = 1.107), ‘overall satisfaction of sessions’ (d = .953), and ‘simulation has made me more aware/worried about clinical practice’ (d = -.947). In contrast, the smallest effect sizes of these differences were obtained for items ‘simulation allows us to plan the patient care effectively’ (d = .238) and ‘the degree of cases difficulty was appropriate to my knowledge’ (d = .257).

**Table 5:** Descriptive data, t-test and effect sizes (d) of differences between two evaluation strategies for each item of clinical simulation satisfaction questionnaire (*n* = 218)

Scale	Formative evaluation (***n*** = 106)	Summative evaluation (***n*** = 112)	t(_**1,217)**_	Sig.	Effect size (d)
	Mean (SD)	Mean (SD)			
1. Facilities and equipment were real	4.41 (0.598)	4.03 (0.963)	4.593	.001	.379
2. Objectives were clear cases	4.47 (0.665)	3.85 (1.125)	14.602	<.001	.623
3. Cases recreated real situations	4.83 (0.425)	4.36 (0.919)	59.431	<.001	.473
4. Timing for each simulation case has been adequate	4.16 (1.025)	3.05 (1.387)	12.403	<.001	1.107
5. The degree of cases difficulty was appropriate to my knowledge.	4.46 (0.650)	4.21 (0.650)	5.138	.013	.257
6. I felt comfortable and respected during the sessions	4.80 (0.486)	4.30 (0.966)	55.071	<.001	.498
7. Clinical simulation is useful to assess a patient’s clinical simulation	4.80 (0.446)	4.18 (0.922)	39.435	<.001	.623
8. Simulation practices help you learn to avoid mistakes	4.83 (0.402)	4.38 (0.903)	77.077	<.001	.446
9. Simulation has helped me to set priorities for action	4.72 (0.530)	4.19 (0.925)	19.479	<.001	.529
10. Simulation has improved my ability to provide care to my patients	4.58 (0.647)	3.87 (1.061)	14.514	<.001	.709
11. Simulation has made me think about my next clinical practice	4.78 (0.478)	4.39 (0.820)	38.654	<.001	.390
12. Simulation improves communication and teamwork	4.69 (0.541)	4.35 (0.946)	27.701	.001	.340
13. Simulation has made me more aware/worried about clinical practice	3.73 (1.231)	4.77 (.849)	12.09	<.001	-.947
14. Simulation is beneficial to relate theory to practice	4.79 (0.407)	4.30 (0.837)	54.177	<.001	.489
15. Simulation allows us to plan the patient care effectively	4.44 (0.677)	4.21 (0.840)	1.055	.022	.238
16. I have improved my technical skills	4.16 (0.758)	3.76 (1.109)	15.460	.002	.401
17. I have reinforced my critical thinking and decision-making	4.41 (0.644)	4.00 (1.048)	7.997	.001	.406
18. Simulation helped me assess patient’s condition	4.48 (0.651)	4.17 (0.994)	6.253	.007	.311
19. This experience has helped me prioritise care	4.63 (0.574)	4.03 (1.035)	19.021	<.001	.605
20. Simulation promotes self-confidence	4.41 (0.714)	3.90 (1.178)	12.818	<.001	.504
21. I have improved communication with the team	4.56 (0.663)	4.29 (0.946)	7.803	.018	.262
22. I have improved communication with the family	2.65 (1.487)	2.77 (1.381)	5.693	.543	-.115
23. I have improved communication with the patient	4.05 (0.970)	3.93 (1.191)	2.187	.420	.119
24. This type of practice has increased my assertiveness	4.40 (0.699)	3.75 (1.234)	25.553	<.001	.649
25. I lost calm during any of the cases	3.09 (1.559)	3.22 (1.559)	.032	.539	-.129
26. Interaction with simulation has improved my clinical competence	4.36 (0.679)	3.81 (1.070)	12.397	<.001	.546
27. The teacher gave constructive feedback after each session	4.79 (0.430)	4.47 (0.880)	43.147	.001	.319
28. Debriefing has helped me reflect on the cases	4.79 (0.492)	4.30 (0.858)	40.809	<.001	.489
29. Debriefing at the end of the session has helped me correct mistakes	4.77 (0.522)	4.21 (0.988)	51.719	<.001	.568
30. I knew the cases theoretical side	4.70 (0.501)	4.33 (0.884)	26.761	<.001	.368
31. I have learned from the mistakes I made during the simulation	4.79 (0.407)	4.39 (0.914)	46.949	<.001	.400
32. Practical utility	4.78 (0.414)	4.15 (1.076)	45.375	<.001	.631
33. Overall satisfaction of sessions	4.92 (0.312)	4.06 (1.016)	79.288	<.001	.953

In addition, participating students provided 74 opinions or suggestions expressed through short comments. Most students’ comments were related to 3 main themes after the thematic analysis: utility of clinical simulation methodology (S45: ‘it has been a useful activity and it helped us to recognize our mistakes and fixing knowledge’, S94: ‘to link theory to practice is essential’), to spend more time on this methodology (S113: ‘I would ask for more practices of this type‘, S178: ‘I feel very happy, but it should be done more frequently’), and its integration into other subjects (S21: ‘I consider this activity should be implemented in more subjects’, S64: ‘I wish there were more simulations in more subjects’). Finally, students´ comments about summative evaluation sessions included other 2 main themes related to: limited time of simulation experience (S134: ‘time is short’, S197: ‘there is no time to perform activities and assess properly’) and students´ anxiety (S123: ‘I was very nervous because people were evaluating me around’, S187: ‘I was more nervous than in a real situation’).

## Discussion

The most significant results obtained in our study are the nursing competency acquisition through clinical simulation by nursing students and the different level of their satisfaction with this methodology depending on the evaluation strategy employed.

Firstly, professors in this study verified most students acquired the nursing competencies to resolve each clinical situation. In our study, professors verified that most nursing students performed the majority of the nursing activities required for the resolution of each MAES© session and OSCE station. This result confirms the findings in other studies that have demonstrated nursing competency acquisition by nursing students through clinical simulation [34, 35], and specifically nursing competencies related to critical patient management [9, 36].

Secondly, students’ satisfaction assessed using both evaluation strategies could be considered high in most items of the questionnaire, regarding their mean scores (quite close to the maximum score in the response scale of the satisfaction questionnaire). The high level of satisfaction expressed by nursing students with clinical simulation obtained in this study is also congruent with empirical evidence, which confirms that this methodology is a useful tool for their learning process [6, 31, 37–40].

However, satisfaction with clinical simulation was higher when students were assessed using formative evaluation. The main students’ complaints with summative evaluation were related to reduced time for performing simulated scenarios and increased anxiety during their clinical performance. Reduced time is a frequent complaint of students in OSCE [23, 41] and clinical simulation methodology [5, 6, 10]. Professors, registered nurses, and clinical placement mentors tested all simulated scenarios and their checklist in this study. They checked the time was enough for its resolution. Another criticism of summative evaluation is increased anxiety. However, several studies have demonstrated during clinical simulation students’ anxiety increase [42, 43] and it is considered as the most disadvantage of clinical simulation [1–10]. In this sense, anxiety may influence negatively students’ learning process [42, 43]. Although the current simulation methodology can mimic the real medical environment to a great degree, it might still be questionable whether students´ performance in the testing environment really represents their true ability. Test anxiety might increase in an unfamiliar testing environment; difficulty to handle unfamiliar technology (i.e., monitor, defibrillator, or other devices that may be different from the ones used in the examinee’s specific clinical environment) or even the need to ‘act as if’ in an artificial scenario (i.e., talking to a simulator, examining a ‘patient’ knowing he/she is an actor or a mannequin) might all compromise examinees’ performance. The best solution to reduce these complaints is the orientation of students to the simulated environment [10, 21–23].

Nevertheless, it should be noted that the diversity in the satisfaction scores obtained in our study could be supported not by the choice of the assessment strategy, but precisely by the different purposes of formative and summative assessment. In this sense, there is a component of anxiety that is intrinsic in summative assessment, which must certify the acquisition of competencies [10–12, 21]. In contrast, this aspect is not present in formative assessment, which is intended to help the student understand the distance to reach the expected level of competence, without penalty effects [10–12].

Both SBA strategies allow educators to evaluate students’ knowledge and apply it in a clinical setting. However, formative evaluation is identified as ‘assessment for learning’ and summative evaluation as ‘assessment of learning’ [44]. Using formative evaluation, educators’ responsibility is to ensure not only what students are learning in the classroom, but also the outcomes of their learning process [45]. In this sense, formative assessment by itself is not enough to determine educational outcomes [46]. Consequently, a checklist for evaluating students’ clinical performance was included in MAES© sessions. Alternatively, educators cannot make any corrections in students’ performance using summative evaluation [45]. Gavriel [44] suggests providing students feedback in this SBA strategy. Therefore, a debriefing phase was included after each OSCE session in our study. The significance of debriefing recognised by nursing students in our study is also congruent with the most evidence found  [13, 15, 16, 47]. Nursing students appreciate feedback about their performance during simulation experience and, consequently, debriefing is considered as the most rewarding phase in clinical simulation by them  [5, 6, 48]. In addition, nursing students in our study expressed they could learn from their mistakes in debriefing. Learn from error is one of the most advantages of clinical simulation shown in several studies  [5, 6, 49] and mistakes should be considered learning opportunities rather than there being embarrassment or punitive consequences  [50].

Furthermore, nursing students who participated in our study considered the practical utility of clinical simulation as another advantage of this teaching methodology. This result is congruent with previous studies [5, 6]. Specifically, our students indicated this methodology is useful to bridge the gap between theory and practice [51, 52]. In this sense, clinical simulation has proven to reduce this gap and, consequently, it has demonstrated to shorten the gap between classrooms and clinical practices  [5, 6, 51, 52]. Therefore, as this teaching methodology relates theory and practice, it helps nursing students to be prepared for their clinical practices and future careers. According to Benner’s model of skill acquisition in nursing [53], nursing students become competent nurses through this learning process, acquiring a degree of safety and clinical experience before their professional careers [54]. Although our research indicates clinical simulation is a useful methodology for the acquisition and learning process of competencies mainly related to adequate management and nursing care of critically ill patients, this acquisition and learning process could be extended to most nursing care settings and its required nursing competencies.

### Limitations and future research

Although checklists employed in OSCE have been criticized for their subjective construction [10, 21–23], they were constructed with the expert consensus of nursing professors, registered nurses and clinical placement mentors. Alternatively, the self-reported questionnaire used to evaluate clinical simulation satisfaction has strong validity. All simulated scenarios were similar in MAES© and OSCE sessions (same clinical situations, patients, actors and number of participating students), although the debriefing method employed after them was different. This difference was due to reduced time in OSCE sessions. Furthermore, it should be pointed out that the two groups of students involved in our study were from different course years and they were exposed to different strategies of SBA. In this sense, future studies should compare nursing students’ satisfaction with both strategies of SBA in the same group of students and using the same debriefing method. Finally, future research should combine formative and summative evaluation for assessing the clinical performance of undergraduate nursing students in simulated scenarios.

## Conclusion

It is needed to provide students feedback about their clinical performance when they are assessed using summative evaluation. Furthermore, it is needed to evaluate whether they achieve learning outcomes when they are assessed using formative evaluation. Consequently, it should be recommended to combine both evaluation strategies in SBA. Although students expressed high satisfaction with clinical simulation methodology, they perceived a reduced time and increased anxiety when they are assessed by summative evaluation. The best solution is the orientation of students to the simulated environment.

## Data Availability

The datasets analysed during the current study are available from the corresponding author on reasonable request.
